# Making space for experiential knowledge in climate change adaptation? Insights from municipal planning officers in Bohol, Philippines

**DOI:** 10.4102/jamba.v10i1.433

**Published:** 2018-03-27

**Authors:** Sébastien Dujardin, Julie Hermesse, Nicolas Dendoncker

**Affiliations:** 1 Department of Geography, University of Namur, Belgium; 2 Institute for the Analysis of Change in Contemporary and Historical Societies, Université Catholique de Louvain, Belgium

## Abstract

Climate change is a global phenomenon that has multiple local effects on people and places. Yet, climate change knowledge often travels uncomfortably across scales and needs constant re-interpretation as it is applied in different spatial contexts. This requires the examination of how scientific and local knowledge about climate change travel across social systems and shape local meanings and adaptive actions on climate change. Using an interpretive social science analysis of environmental change, this study investigates development planning as a key boundary object for handling both kinds of knowledge and explores experiential knowledge of climate change held by planning officers from the coastal landscape of the island province of Bohol, Philippines. Drawing upon face-to-face interviews, mental maps, and planning documents review, main results first characterise three experiential ways of knowing about climate change across spaces of lived experiences and spaces of maps and plans. Then, we show how planners engage with climate change adaptation by combining national, techno-scientific and local, on-the-ground ways of knowing, offering a venue in which experiential knowledge on climate change is used for building planning significance and making more grounded accounts of adaptation moving forward in planning policy and practice.

## Introduction

### Background

Climate change is perhaps the greatest challenge facing society as it is a global phenomenon that has multiple local effects on people and places. These effects will increase over time and require planning and policy solutions far beyond the future that is usually imagined in the human lifespan and the world of politics. Although historically adaptation planning and policy has focused mainly at the national level (Agrawal 2010; Tompkins 2005), attention to adaptation at the local level has received an increasing interest during the last decade. This emphasis on local adaptation stems from a prevailing opinion in the adaptation literature that ‘adaptation is local’ (Measham et al. 2011:890). The impacts of climate change are experienced locally, and therefore, geographic variability in climate impacts requires ‘place-based’ approaches to climate vulnerability analysis and adaptation (Cutter, Mitchell & Scott 2000; Groulx et al. 2014; Storbjörk 2007).

Integrating local level and scientific knowledge is often highly valued for effectively addressing climate change (Cash & Moser 2000). The Intergovernmental Panel on Climate Change (IPCC), for instance, supports with ‘robust evidence’ that the ‘integration of local knowledge with additional scientific and technical knowledge can improve disaster risk reduction and climate change adaptation’ (IPCC 2012:15). Scientific knowledge is commonly understood as knowledge generated systematically from formalised, explicit processes and principles, such as scientifically acknowledged methods and theories. Local knowledge, in turn, often refers to a broad set of knowledge situated in specific locales that reflects expertise and understanding of local phenomena (Raymond et al. 2010). However, the status of local knowledge in relation to scientific knowledge and its relevance in the face of future climate change remains contested (Adger et al. 2009). Bringing together and making use of local and scientific knowledge is not straightforward (Kristjanson et al. 2009) and it is often difficult for individuals and organisations to handle both kinds of knowledge.

As a response, this article aims at exploring the multiple understandings of climate change by enlisting the conceptual lens of experiential knowledge to examine how scientific and local knowledge about climate change travel across social systems and shape local meanings and actions on climate change within the field of development planning. Within the next sections, we start by addressing the multiple understandings of climate change and introducing the concept of experiential knowledge. We then present the adaptation and development planning context of our case study, the island province of Bohol (Philippines), as well as our data collection approach and interpretational strategy. Furthermore, we present our main results, including the characterisation of three experiential ways of knowing about climate change. Lastly, we detail and discuss how planners build planning significance by combining both local and scientific knowledge to manage the future of their coastal landscape.

## The multiple understandings of climate change and experiential knowledge

Climate change has multiple and contradictory understandings. It is a scientific and conceptual term that draws upon a diverse range of data and observations that may be considered spatially and temporally relative as well as culturally or geographically specific (Yager 2015). Although it has gained a global recognition during the last decade, climate change is a statistical assemblage of average weather over a 30-year period that presents ambiguities, including within incompatible definitions from the IPCC and United Nations Framework Convention on Climate Change (UNFCCC) (see Brace & Geoghegan 2010). Yet, viewing climate as a composite of local climate quantities ‘wholly disembodied from its multiple and contradictory cultural meanings’ (Hulme 2008:6) cannot accommodate the ways in which it may be understood ‘through cumulative sensory experiences, mental assimilation, social learning and cultural interpretations’ (Hulme et al. 2009:197). Understandings of climate change in one community or society are often different from those in another.

Besides, while scientific knowledge from IPCC assessments exerts considerable hegemony over the climate change debate (Hulme & Mahony 2010), scientific knowledge that is claimed by its producers to have universal authority is received and interpreted very differently in different political and cultural settings. For instance, the consensus science of the IPCC might look persuasive from the centralised sites of production, and the global knowledge that it produces ‘helps governments erect and then justify their simplified constructions of people and nature, and the institutions based on them’ (Fogel 2004:109). However, the views from the peripheries of space, power and culture – the very places where knowledge is consumed for developing adaptive capacity and action – look very different. As Adger et al. (2009) showed, reactions to climate change and rising sea levels are embedded in much wider stories about the economic, cultural and demographic aspirations, and explain why universal and globalised constructions of climate and adaptation often fail to connect with the local.

Climate change knowledge therefore often travels uncomfortably across scales and needs constant re-interpretation as it is applied in different spatial contexts. Such a re-interpretation, we suggest, may be revealed by exploring shared knowledge sets and experiences. Indeed, the recent literature has tended to support a binary between scientific and local knowledge, seeing science as a groundless set of ideas, and ignoring the hybrid forms combining various scientific and local knowledges that emerge in the actual practice of everyday life. Nonetheless, as Agrawal (1995:30–31) puts it, ‘it makes much more sense, […] to talk about multiple domains and types of knowledges, with differing logics and epistemologies’. In response, this article follows Rice, Burke and Heynen’s (2015) engagement for new forms of praxis-oriented research that considers situated forms of knowledge, fully accounting for the epistemologies and experiences through which people come to know climate change.

In particular, we consider experiential knowledge as a hybrid form of knowledge involving both the local and scientific domains. First coined by the Nobel laureate economist F.A. Hayek, experiential knowledge includes, for instance, the practical knowledge gained through practicing a profession such as farming, or logging, or fishing (Karl et al. 2012). Yet, we argue, it can also involve any practical knowledge coming from beyond local and professional experiences. Derived from one’s own life experience, experiential knowledge ‘enables us to ask how a variety of publics make sense of climate change, as witnessed and responded to in ordinary, everyday-life scenarios’ (Brace & Geoghegan 2010:289). It is often tacit or implicit and thus usually not formalised or systemised. Drawing upon this conceptual lens of experiential knowledge we explore in detail one important but neglected area: discourses of climate change held by social actors involved in development planning practices.

## Development planning and climate change knowledge

Understood as place-based problem-solving aimed at sustainable development (Davoudi et al. 2009), development planning involves processes through which options for the development of places are envisioned, assessed, negotiated, agreed and expressed in development policy terms. A central element within these processes is the orchestration of multiple types of viewpoints and knowledge. In this sense, we consider development planning as a boundary object having a key role to play for bringing local and scientific understandings of climate change together. Development planning allows for response to Wenger’s (2000) call for organisations, individuals or tools that can work across this epistemic divide.

Within the field of development planning, planners are key social actors for building a set of approaches that allow applying knowledge to action (Friedmann 1987) or simply ‘figuring out what needs to be done and how to do it’ (Randolph 2004:16). Planners’ situated knowledge and awareness of local sensitivities, including the interactions between biophysical and social contexts, is important in understanding local phenomena, balancing competing priorities and values, policy making and managing socio-ecological systems (Berkes & Folke 2002; Picketts et al. 2012). They can be viewed as key stakeholders for bridging the gap between top-down and bottom-up actions.

Planners’ access to various sets of information and knowledge allow them, for instance, to understand how potential adaptation strategies may conflict with other locally significant social goals and preferences (Adger et al. 2009) or bridge the gap in disaster risk reduction between top-down and bottom-up actions (Gaillard & Mercer 2013). This involves accessing both scientific and local information coming from professional experience, familiarity with place, and practical knowledge of resource management traditions, tools, techniques and technologies.

However, poor evidence exists about how planners perceive, understand and engage with climate change. In particular, little is known on how local climate change knowledge is made and modified in the context of development planning institutions, especially in relation to planners’ knowledge sets and experiences. While most research focuses on the ways actors and institutions can better mainstream climate change adaptation into local policy and practice through the integration of climate change into local development plans (see, for instance, Hedger et al. 2011; Lasco et al. 2009), the literature lacks evidence showing how local actors from the municipal level perceive climate change and the adaptation challenge in their daily practice of land use management.

This article therefore draws on and develops the recent interest in climate change from cultural geographers, anthropologists, social scientists and others, much of which argues for a more grounded and localised understanding of climate change informed by a spatially contingent view of knowledge (Barnes & Dove 2015; Betsill & Bulkeley 2007; Hulme 2008; Reuter 2015). It aims at soliciting and valuing experiential knowledge from social actors within the field of development planning, and transcending the narrow process of mainstreaming climate change adaptation into development planning and the climate debate more generally. By exploring local planners’ discourses on climate change through an interpretive social science analysis of environmental change, the specific aim of this study is twofold: (1) to describe how planners engage with the multiple ways of knowing climate change and (2) to identify the types of knowledge involved in these accounts and their spatial orderings. In this sense, we support a deeper engagement with experiential knowledge for understanding adaptation strategies, planning policy and challenges.

## Climate change adaptation and local development planning in Bohol, the Philippines

Climate change poses a unique set of challenges to archipelagic countries such as the Philippines where coastal areas are often considered as the most vulnerable to the adverse impacts of climate change (Rincón & Virtucio 2008). Seventy per cent of the country’s 1500 municipalities are located along the shoreline, and most of the people are dependent on the coastal ecosystem for their livelihood (Capili, Ibay & Villarin 2005). Such a situation poses serious challenges for sustainable land use and management. In response, numerous climate change adaptation strategies are being undertaken by multiple types of social actors (AKP 2012), including the national government that has passed the *Climate Change Act* in 2009 in order to implement climate compatible development at the local level.

In this context, the island of Bohol was designated as a pilot province for the project named ‘Integrating Disaster Risk Reduction and Climate Change Adaptation in Local Planning and Decision-making Processes’ (Cañares 2012), which makes local governments additionally solicited to address the challenge of climate change in local development planning. As a practical implication, municipal planning and development offices in Bohol have been endorsed to take responsibility for addressing adaptation in the elaboration of local development plans, including strategic ones such as the Comprehensive Land Use Plan (CLUP).

The CLUP is one of the fundamental tools for local governance and decision-making in the Philippines. Specifically, the document is a policy guide for the regulation of land uses within local governments’ territorial jurisdiction. It defines the policies on settlements, protected and production areas and infrastructure that should be consistent with the overall development vision. As the CLUP has a time frame of 10 years, municipalities in Bohol are progressively considering the new requirement of integrating climate change adaptation when the plan expires and needs to be reviewed. Within this process, municipal planning and development offices are playing an essential role as coordinators in the crafting of the plan.

Although some authors pointed that levels of knowledge and awareness on climate change impacts, mitigation and adaptation measures in South-East Asia are low among local officials and insufficient to prompt them to formulate proactive action agendas (Sales 2008), this study builds upon Cañares’ (2012) work on local governance in Bohol that showed the recent increase of climate change awareness among local government officers because of both the newly approved legislation and the mainstreaming project on climate change adaptation.

In addition, as Resurreccion, Sajor and Fajber (2008) highlighted, local governments have been traditionally responding to climate change extreme events. Within disaster-prone municipalities of Bohol, this falls under the conventional mandate of preparing disaster risk reduction and management (DRRM) plans. Local governments thus already have a tangible amount of practical knowledge on how to address climate-related risks, which provides an additional opportunity for exploring social actors’ multiple ways of knowing about climate change.

## Data collection

**FIGURE 1:**
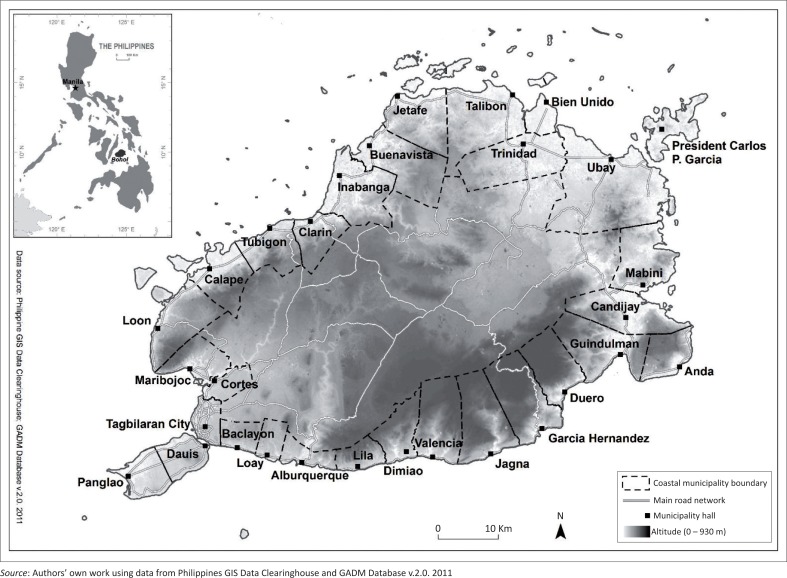
Location of Bohol within the Philippines and coastal municipalities surveyed.

In order to investigate local discourses and experiences on climate change, this study builds upon a number of sources, including preconstructed and self-constructed data (Cloke et al. 2004). The main self-constructed data set consists of in-depth face-to-face interviews conducted between June and September 2013 with Municipal Planning and Development Coordinators (MPDCs) from the 30 coastal municipalities of Bohol (see Figure 1). Development planning officers play an important role in the management and protection of community resources through regulation of land development, building codes, transportation and environmental-related issues. Their experience can be explored as a ‘knowledge-in-practice’ and ‘on-the-ground’. Focusing on coastal municipalities allows reflecting the diversity in terms of size, demographic profile and location, as well as social vulnerability and adaptive capacity to different climate-related hazards.

A total of 29 planning officers took part in the interviews (one officer dismissed the interview request). Most of them were male officers (21), with a working experience as a head of the office ranging from a couple of months (officers newly appointed) up to 32 years. MPDCs’ median number of years’ experience reaches 20 when considering their total experience within the municipality (either as a member of the planning office or as an officer from another department such as the Municipal Agricultural Office, for instance). This shows how much most interviewees have performed and capitalised a substantial amount of developmental work within their institution.

All interviews took place on site at Municipality Halls, within municipal planning and development offices and lasted between 30 min and 1 h 15 min. Interviews focused on planning officers’ perceptions of local climate change and associated risks and the potential role of local development planning in addressing the adaptation challenge. They were guided by a list of key questions that allowed an in-depth exploration of MPDCs’ multiple ways of understanding and engaging with climate change:

- Do you perceive that ‘climate change’ is happening in your municipality?
- To what extent are people affected and/or requesting for interventions?
- What can your office do to address climate change?
- Do you have specific plans or programmes?
- What role does the CLUP play in addressing climate change?

As first interviews were being conducted, it quickly became essential to explain that this study was not aimed at evaluating informants’ activities or assessing the extent to which climate change adaptation had been mainstreamed into development planning so far. Likewise, it was not essential to test whether informants’ observations can be assessed for accuracy or veracity compared to other sets of scientific knowledge or measurements previously gathered in the literature review. Rather, it was more relevant to ask how planning officers are making sense of ‘climate change’ as both a physical and intellectual artefact in the everyday spaces they manage.

Interviews took place with the support of a simplified map of the municipality displaying *barangays*’ names and boundaries (i.e. the smallest political administrative division, which stands under the municipal level), river and road networks and topographical contour lines. Interviewees were invited to locate on the map the critical places mentioned when describing the impacts of climate change observed within their municipality. Mainly used in behavioural geography for the study of environmental perceptions, mental maps allowed surveying the spatial cognitive representation (Freundschuh 1992) of climate-related risks and characterising planning officers’ territorial representations of adaptive measures.

## Interpretational strategy

For the purpose of this study, we embraced what Cloke et al. (2004) describe as the artisan figure or researcher. Through a detailed and close reading of interview transcripts, this interpretational strategy provides a mode of understanding planners’ world views as it is revealed in their everyday experience, encounters and utterances. Drawing upon the best known and most widely applied grounded theory (Glaser & Strauss 1967), it follows a set of procedures that offer clear guidelines for practice, without being overly rigid or prescriptive. Throughout the analysis, both manifest (content) and latent (discourses) data were considered. Categorisation and theoretical constructions evolved simultaneously in a two-way relationship. Oscillating between these two approaches thus became particularly relevant as informants’ realities were analysed as embodied performances of broader social and institutional discourses.

As a way to prepare interviews, and also for performing triangulation with the information discussed with planning officers, nine complete versions of CLUP were collected from the municipal and provincial planning and development offices. Along with interview transcripts, the CLUPs were subject to interpretive content analysis, which also drew on the artisanal interpretive strategy. By tackling planners’ discourses and experiences on climate change through face-to-face interviews, mental maps and document analysis, we gained a measure of heterogeneity within our field of analytic vision. The following result sections explore planners’ experiential ways of knowing climate change through this diversity of research material.

## Planners’ experiential ways of knowing climate change

An important diversity of answers was provided when addressing the idea of ‘climate change’ with planning officers from the coastal municipalities of Bohol. These ranged from reporting and describing climate-related hazards experienced within the municipality to enumerating adaptive measures currently undertaken by the planning and development office, along with personal perceptions of change in the weather and seasons. As the material was coded according to the framework suggested by Cloke et al. (2004), the following patterns emerged: planning officers consider climate change simultaneously as (1) a *reality* they observe on a daily basis, (2) a *problem* they should address through their planning activities and (3) an *agenda* affecting their tools and practices (see Table 1). While this betrays what Brace and Geoghegan (2010:2) describe as the ‘definitional ambiguity’ of climate change, it also shows that planners relate to climate change by mobilising hybrid forms of knowledge involving various sets of experiences. We successively detail how these three ways of knowing about climate change play out as experiential knowledge in planning officers’ narratives about past, present and future development and planning practices.

**TABLE 1:** Summary of planners’ experiential ways of knowing about climate change within the coastal municipalities of Bohol.

Experiential way of knowing about climate change	Main evidence provided by planning officers
Reality	The weather has become eratic (unseasonal rains, high-heat days)
	Municipalities experience abnormal tides (coastal and estuarine areas)
	Unexpected extreme events are increasing (heavier rainfalls, stronger Habagat [monsoon] winds, bigger waves or storm surges)
Problem	Greater disaster risks resulting from flood, landslides, sea level rise, droughts, coastal erosion, storms and typhoons
	Negative impacts on livelihoods (e.g. lower yields and catches for farmers and fishers, respectively)
	Land use change (e.g. storm surges and sea salt intrusions lead to the conversion of ricefields into mangrove plantations)
Agenda	Climate change has become part of planners’ mandate and duties since the *2009 Climate Change Act*
	Recent attendance to training programs led by the Housing and Land Use Regularatory Board in Cebu
	Current planning activities focused on the integration of CCA into the CLUP via the DRRM plan and geo-hazard maps

CLUP, Comprehensive Land Use Plan; DRRM, disaster risk reduction and management; CCA, Climate Change Adaptation; DRRM, Disaster Risk Reduction and Management.

### Planners’ observations of a changing climate

Firstly, planning officers reported multiple indications that climate change has become part of their daily reality. They described several observations supporting the idea of a changing climate, including erratic weather patterns, higher tides and unexpected extreme events. Many informants feel the pattern of rainy seasons is not the same as before, although they acknowledge lacking ‘formal’ evidence to support it. As one MPDC explains^1^:

We have no data yet […] which can support the effects of climate change. But as far as we all know, it is really happening. The municipality is already affected. And part of it, I can say, it is because the weather is not the usual one.

Many informants perceive that the weather has become erratic because of the increasing occurrence of unseasonal rains and high-heat days throughout the year. They reported experiencing unexpected rains during the dry season, and very hot days during the rainy season. Besides, many planners stated that the start of the rainy season has become unpredictable although it is usually expected around June or July.

Another observation of climate change narrated by planning officers is the occurrence of unexpected extreme weather events such as heavier rainfalls, stronger monsoon winds and bigger waves during storms or typhoons. In this regard, some believe that the occurrence and track pattern of tropical cyclones have become unpredictable. Along with these indications of weather variability, another change evoked by planning officers is the increase of tidal heights affecting low lying areas such as coastal, estuarine and small island *barangays*. They reported higher sea water levels than usual, especially when high tides and low-pressure weather conditions are combined.

These observations of sea level change and weather variability echo Sarewitz and Pielke’s (2000:56) idea that people cannot directly sense global warming as socio-ecological systems experience local and regional temperatures, not the global average. Yet, ‘climate has myriad facets to which humans could attune’ (Sarewitz & Pielke 2003:246). Therefore, although weather variability and sea level change are not the same as climate change, and not necessarily direct proxies for it, these remain the phenomena to which most planning officers referred to as a way of narrating climate change. Planning officers’ understanding of climate change might thus not be measurable or backed up with formal, scientific data, but are realities around which they build a narrative. Within the next section, we detail how planners relate their observations of a changing climate with their experiences of climate-related issues.

### Planners’ experiences of climate-related issues

**FIGURE 2:**
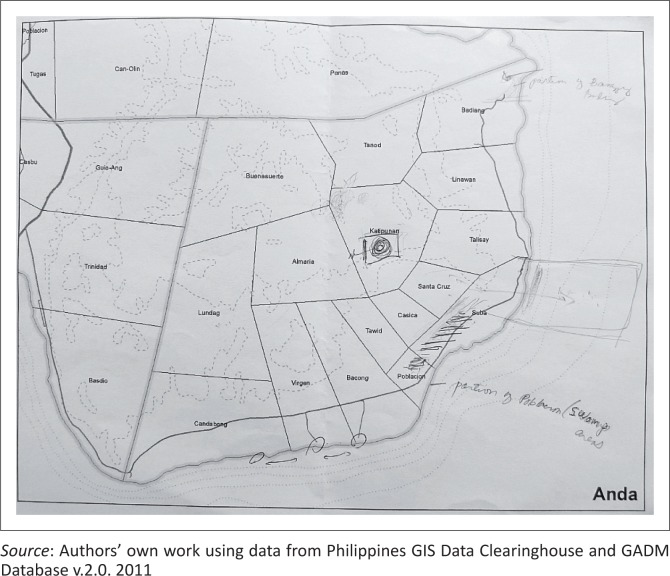
Example of a mental map picturing one informant’s interpretation of flood risks within the coastal *barangay* of Suba.

Secondly, planning officers are also making sense of climate change as a source of problems in the everyday space they manage. They are observing multiple local ‘manifestations of climate change’ in the context of their professional expertise in planning and development. Their experiential knowledge of climate change emerges from two types of interrelated planning practices: addressing developmental issues and managing disaster risks. Planning officers drew upon those relational activities to provide evidence about the climate-related issues faced within their municipality. Using mental maps as a means of representation (see example in Figure 2), they provided geographically situated and locally grounded accounts of such evidence.

As first major evidence, planning officers highlighted the increased occurrence of flood events affecting areas located along the sea and rivers, including within both built and non-built environments (e.g. coastal roads, city centres and rice paddies). Estuarine areas were often designated as the most critical places because of a double exposure when the events of high tides and high river stream flow resulting from heavy rains combine. A second evidence of climate change provided by planning officers was the impacts of sea level rise on the coastal areas, especially small island *barangays*. As one MPDC from the northern part of the island stated, the ‘abnormalities of sea water levels’ cause the recurrent submersion of rice paddies, mangroves, roads and houses located close to the sea. Interrelated with this issue is coastal erosion, which is viewed as a threat for properties along the coastline. Along with flood and sea level rise issues in the lowlands, MPDCs also mentioned landslides in the uplands as being part of the evidence of climate change. More frequent heavy rains and the resulting high volumes of water affect settlements and obstruct *barangay* road networks, especially on steep slopes areas.

Along with referring to disaster risks as proxies of climate change impacts, planning officers perceive weather variability and sea level rise as agents of change undermining the local pursuit of livelihoods. They reported fishermen and famers as being the most affected by climate change. As one MPDC who used to farm claims, farming has become a risky activity because ‘farmers have to gamble with the weather’. Unpredictable rainy seasons, heavy rain, droughts and salt intrusions are pointed to be responsible for generating lower yields for crops such as rice, corn and other root vegetables (e.g. *camote, ubey*). Regarding fishing activities, the change in sea temperatures and stronger winds are associated with coral reef degradation and lower catches, while sea level rise is causing the overflowing of dikes and loss of breed fishes.

As a response to these climate-related issues, MPDCs reported changes in socio-spatial practices. In the case of sea level rise and salt intrusion of rice paddies, several MPDCs reported that farmers are converting their rice paddies into mangrove plantations. As one MPDC whose municipality contains reef islets claims, households from small island *barangays* are building their houses higher ‘to adapt to the rising sea level’. For coastal land owners on the main island, those who can afford it are building sea walls, including in front of resort developments, for instance. Land use conversion and changes in building practices are thus autonomous forms of adaptation undertaken on the basis of individuals and communities’ lay understandings of climate change. These represent a form of local knowledge that planning officers ‘upscale’ and integrate as part of their professional experience about climate change within their municipality.

Meanwhile, MPDCs also provided personal accounts of climate-related risks when explaining their own experience of climate-related issues, that is, when they were either personally affected by a disaster, or at the forefront of disaster relief. One MPDC, for instance, emotionally recalled being affected by floods when living in a flood-prone area with her family^2^:

When heavy rains come and the tide is high, the portion of [*barangay*] Suba facing the mountains is really affected. The sea waters will go through houses […]. I experienced it. That was a rainy day. Three days raining with high tides. Then water came into our dining room and rose up to the knee level. But I left the house since. I decided I will no longer experience this.

This narrative suggests that the coastal spaces managed by planning officers are also places of embodied experience where they make sense of what past and future weather variability and sea level change feel like.

This type of embodied knowledge is further built by planning officers’ experiences of coordinating disaster response activities. As one MPDC also in charge of DRRM explained in an agitated way:

Yesterday night we experienced strong winds. We had ‘ipo-ipo’, a small tornado. Here in one of our town. Some houses lost their roof. Blown away! But luckily there were only small injuries. This is why I am arranging some cleaning operations for the branches that fell off the trees.

Within these accounts, boundaries between personal and professional experiences are blurred. Planning officers’ experiential knowledge of climate change is built upon a combination of both personal, embodied experiences of weather variability and professional experiences of dealing with developmental issues and the managing disaster risks. These provide them with grounded sense of what climate’s material effects feel like, and involves their duty-based responsibilities. Within the next section, we describe how this experiential knowledge connects with planners’ tools and practices.

### Planners’ tools and practices

Thirdly, planning officers understand climate change as an agenda. Their accounts of climate change also draw upon a law requirement from the *2009 Climate Change Act* that sets climate change at the planning and development agenda. In particular, planners’ experiential way of knowing climate change is built upon their duty-based responsibility of integrating climate change adaptation into local development plans, including the municipal Comprehensive Land Use Development Plan. The latter is viewed by MPDCs as an important planning tool for addressing climate-related risks. Qualified by an informant as ‘do’s and don’ts’, it allows determining ‘climate risk-free areas’, and defining zones where development can and cannot be located. As a means of integrating climate change adaptation into local development plans, planning offices consider integrating the municipal DRRM plans into the revised CLUPs as essential.

Specifically, MPDCs strongly rely upon the integration of geo-hazards maps developed by the Mines and Geosciences Bureau of the National Department of the Environment and Natural Resources. Drawing upon base maps, satellite imagery and fieldwork, the 1:50.000-scale geo-hazard maps classify areas as either low, moderate or highly susceptible to hazards such as floods, landslides and storm surges. As one MPDC explained:

Hazard maps are produced by some government agencies as to the vulnerability of our LGU^3^ to climate change hazards. They have conducted some surveys and made this kind of map that reflects storm surges areas.

This account of maps’ origins highlights that planners’ experiential knowledge on climate change is also informed by scientific knowledge coming from the highest level of the planning hierarchy. Such a way of knowing about climate change to manage municipal lands answers Hulme (2008:8)’s call for a better understanding of ‘the spatial ordering of climate change knowledge’ in the specific context of local development planning.

Within the Filipino planning system, national government agencies are the producers of scientific knowledge. The latter is generated by technical experts at the national level and consumed by local planning officers at the municipal level for determining hazard prone areas. In the province of Bohol, such a propagation of scientific knowledge downstream has been facilitated by the national government-led program called READY (Hazards Mapping and Assessment for Effective Community-Based Disaster Risk Management), which involved conducting municipal training about mainstreaming DRRM into local development processes. Hazard maps are thus planning tools carrying climate-related knowledge that flows smoothly between the central production of scientific knowledge and its periphery. Within the last result section, we show that one-way local knowledge and scientific knowledge combine to form a key element in the process of building planning significance.

## Building planning significance through local and scientific ways of knowing

Planners’ three main experiential ways of knowing about climate change suggest the old-fashioned idea that ‘scientific facts build the appropriate foundation for knowing how to act in the world’ (Sarewitz 2004:385) needs to be challenged. Indeed, planning officers’ observations of a changing climate, their experiences of climate-related issues and their accounts of geo-hazard maps show how scientific and local ways of knowing about climate-related risks can develop planning significance through their interactions. Derived from scientific knowledge, most planning officers view geo-hazard maps as a taken-for-granted spatial ordering of climate risks designating ‘which areas are free for development’. Yet, some informants also raised the issue of scale inherent to this kind of planning document as it only provides ‘a general description of the type of hazards’, and is not always easily applicable at the *barangay* level. As a way to compensate the coarse resolution of hazard maps, one MPDC, for instance, referred to his field experience telling that:

We can also locate on the map where the areas prone to flood hazards are. We can, because we know the vicinity. Like, for example, the risk of flash floods in Poblacion. Then, we have a flood prone area in Nahawan because of the river which is on the border with Inabanga Municipality. During heavy rainfalls up hill, the river will overflow and affect up to 80 households.

Planning officers’ local knowledge of climate risks thus supports them in the exercise of building contingency plans. It allows completing the techno-scientific knowledge brought about by geo-hazard maps regarding municipalities’ level of climate exposure and vulnerability.

These kinds of place-based, non-scientific ways of knowing often rest on planning officers’ sense of place. In the case of exposure to coastal floods, for instance, MPDCs referred to their municipality’s specific geographical context such as being part of the Maribojoc bay, or being protected by neighbourhood islands such as Panglao or the islets from the northern double coral reef barrier (see Figure 1). Further evoking the etymology of his municipality’s name, one MPDC emphasised the city’s low historical record of coastal floods. She explained that the municipality name of Tagbilaran comes from the word *Tinabilan*, meaning ‘screened’ or ‘shielded’, by reference to the fact that the city is protected on the southwest from the open sea and strong monsoon winds by the nearby island of Panglao. This kind of understanding of municipalities’ physical landscapes supports planning officers’ sense of place and allows them to build planning significance by combining both scientific and local ways of knowing.

Scientific and local forms of knowledge such as hazard maps therefore should not be viewed as mutually exclusive for implementing planning policies. On the contrary, the Bohol case study shows how both forms of knowledge contribute jointly to place-based adaptation and complete other studies undertaken outside the field of planning that value the potential role of experiential knowledge (e.g. Laidler 2006; Orlove et al. 2010). Investigating planners’ multiple ways of knowing climate change does not mean valuing local knowledge more than scientific knowledge. Instead, this research advocates for approaches focusing on the ways that scientific and local ways of knowing can develop planning significance through their interactions. Ultimately, it calls for a greater understanding of how development planners might use and combine local knowledge from their relational practices with scientific knowledge as brought about by institutional processes for acting on climate change.

## Conclusion

Climate change is a global phenomenon that has multiple local effects on people and places. This requires examination of how scientific and local knowledge about climate change travel across social systems and shape local meanings and adaptive actions on climate change. Using an interpretive social science analysis of environmental change, this study investigated development planning as a key boundary object for handling both kinds of knowledge, and explored experiential knowledge of climate change held by local planning officers from the coastal landscape of the island province of Bohol, the Philippines. Soliciting and valuing local perceptions of climate change allowed for greater attention to the specificities of planners’ experiences of climate and place, a re-evaluation of local knowledge and a novel understanding of the ways in which local planners deal with multiple types of knowledge to manage the future of a coastal landscape.

Drawing upon face-to-face interviews, mental maps and planning documents review, we described three experiential ways of knowing about climate change (i.e. as reality, a problem and an agenda) across spaces of lived experiences and spaces of maps and plans. First, we highlighted how planning officers perceive climate change as a reality through the manifestation of weather variability and sea level change. Then, we laid down planners’ sets of experiences with climate-related issues, including disaster risks and the ways these affect local socio-spatial practices. Lastly, we showed the way planners address climate change through their duty-based responsibility of integrating climate change adaptation into local development planning, especially by integrating geo-hazard maps into strategic municipal land use development plans.

Results highlight ‘the spatial ordering of climate change knowledge’ (Hulme 2008) in the context of local development planning. We show that climate change has worked its way into a set of discourses that are beyond climate knowledge as brought about by institutional processes, highlighting that local sets of experiences also offer a venue in which knowledge on climate change is made and used for building planning significance. Planners’ local understanding and engagement with climate change draw upon a combination of personal, embodied experiences of weather variability and a set of professional experiences dealing with developmental issues and addressing disaster risks. These provide them with grounded ways of knowing what climate change feels like and involves regarding the management of their municipal space. Such a type of experiential knowledge combines planners’ local, on-the-ground knowledge with the national, techno-scientific knowledge from higher levels of the planning hierarchy, which supports planners’ duty of assessing local vulnerabilities and building contingency plans.

Planners’ experiential way of knowing and addressing climate change therefore offers a venue for making more grounded accounts of climate change adaptation moving forward. It suggests that local government institutions may act upon climate change by using the context-specific knowledge they already have without necessarily relying upon detailed climate models. In practice, planning officers can play a key role as ‘co-creators’ of climate knowledge by, for instance, combining the techno-scientific information coming from national agencies and local adaptive practices from most vulnerable individuals and communities. In this way, making space for experiential knowledge promotes planning outcomes that are grounded into a ‘home made’ or ‘produced and consumed at home’ knowledge on climate change. It becomes an asset for integrating the diversity of knowledge needed to develop inclusive planning practices and implement adaptation strategies.

Turning planning officers’ experiential knowledge into action, however, also reminds us Inderberg’s (2014:13) observation that adaptation is a political process where different agendas (not always transparent) are involved and affected. The Bohol case study investigated here did not consider the political dimension of local development planning for bridging planners’ experiential knowledge and the implementation of planning policies. Indeed, although municipal planners identified some strategic measures as critical and urgent (e.g. building sea walls or relocating residents from flood-prone areas), if these are not supported by the priorities from the political component of the local government, they will hardly be considered and allocated the necessary funds. As Cañares (2012:4) explains, how local development plans are translated into concrete actions and programs are dependent on the ‘prioritisation ethic’ of elected leaders who have the political power to steer the Annual Investment Plan, which is the primary basis of the local government’s annual budget. Bringing this critique along, this research calls for greater attention to the role planners may play as key interested participants, or ‘coalition builders’, engaged in the co-production of knowledges between differing social groups and networks in order to promote integrative development planning processes.

## Footnotes

1 We use the acronym of MPDC for making reference to research informants but do not mention the municipality in order to preserve their anonymity.

2 See mental map of Anda in Figure 2.

3 A Local Government Unit (LGU) in the Filipino context may be a province, a city, a municipality or a barangay in the descending order of geographical scale and scope of political power. In geographical terms, each LGU has its own territorial jurisdiction, but the smaller unit is embedded in the next higher unit, forming a nested pattern. Within this statement, the planning officer refers to the municipal level.
